# Risk factors associated with acute pancreatitis in diabetic ketoacidosis patients: a 11-year experience in a single tertiary medical center and comprehensive literature review

**DOI:** 10.3389/fmed.2025.1571631

**Published:** 2025-04-07

**Authors:** Yifei Chen, Yan Bo, Zhuanzhuan Han, Mengjie Chen

**Affiliations:** ^1^The Department of Emergency Medicine, The Affiliated Hospital of Yangzhou University, Yangzhou, Jiangsu, China; ^2^The Department of Medicine, Northwest Minzu University, Lanzhou, Gansu, China; ^3^The Department of Medicine, Dalian Medical University, Dalian, Liaoning, China

**Keywords:** diabetic ketoacidosis, acute pancreatitis, pancreatitis, risk factors, diagnosis

## Abstract

**Objective:**

To identify independent risk factors and predictive markers for acute pancreatitis (AP) in patients with diabetic ketoacidosis (DKA).

**Methods:**

Here, we reported a 11-year experience in a single tertiary medical center and conduct a systematic review of acute pancreatitis in diabetic ketoacidosis patients (DKA-AP). 1,941 cases were included. First retrospective cohort study analyzed clinical data from 45 patients with DKA-AP and 45 matched controls with DKA alone (DKA-nonAP) admitted to Yangzhou University Affiliated Hospital (2013–2024). Baseline characteristics included BMI, hyperlipidemia history, and serological profiles. Secondly we retrieved clinical studies of DKA-AP from PubMed database system and analyzed these clinical data in depth.

**Results:**

Significant differences were observed in BMI: median [IQR] = 26.30 [22.15–28.40] vs. 23.20 [20.70–25.35] kg/m^2^, *p* = 0.031, hyperlipidemia history (26.7% vs. 4.4%, *p* = 0.004), abdominal pain duration (3 vs. 0 days, *p* < 0.001), and lipid profiles TC: median [IQR] = 7.71 [5.60–11.66] vs. 4.86 [4.22–6.52] mmol/L, *p* < 0.001; TG: median [IQR] = 11.90 [4.71–15.84] vs. 1.60 [0.89–3.64] mmol/L, *p* < 0.001. Logistic regression identified TC (OR = 1.455, 95% CI: 1.196–1.769), TG (OR = 2.046, 95% CI: 1.202–3.484), and abdominal pain duration (OR = 3.892, 95% CI: 2.173–6.972) as independent risk factors. Receiver operating characteristic curve (ROC) analysis demonstrated strong predictive performance (combined AUC = 0.933, sensitivity = 93.3%, specificity = 73.3%). Moreover, we found 11 clinical studies investigating DKA-AP. Cumulatively, 1,851 patients were studied, including 3 interventional studies, 1 genetic observational study, and 7 cohort studies exploring risk factors.

**Conclusion:**

Elevated TC, TG, and prolonged abdominal pain duration are key predictors of DKA-AP. These factors enhance early diagnosis and clinical management in Yangzhou, China.

## 1 Introduction

The current clinical evidence supporting the existence of a distinct set of risk factors for the co-occurrence of diabetic ketoacidosis (DKA) and acute pancreatitis (AP), called DKA-AP, is inconclusive. The interplay between DKA and AP disease makes the clinical diagnosis of the DKA-AP more challenging, which can result in death. Nair and Satheesh have indicated that the incidence of unrecognized DKA-AP accounts for at least 15% of the DKA population and have broadly called for clinicians to screen for DKA-AP by serum amylase, lipase, and triglyceride assessment (1). An analysis of a U.S. population-based survey revealed a higher mortality rate in patients with DKA-AP compared to those with AP (aOR 2.3, *p* < 0.001; CI 1.8–3.0). It is noteworthy that this study demonstrated a national incidence of DKA-AP of 1% in the United States between 2003 and 2013, with a cumulative total of 33,356 patients with DKA-AP (2). The presented data indicates that DKA-AP is not an exceedingly uncommon occurrence. The reason for the failure to diagnose DKA-AP, which has resulted in fatalities among patients with DKA or AP, may be attributed to the absence of validated clinical predictive risk factors upon which to base clinical diagnostic decisions. The objective of this study was to examine the risk factors associated with DKA-AP through a retrospective analysis and to evaluate the predictive efficacy of the identified risk factors. This will contribute to the prevention and management of patients with DKA-AP and enhance diagnostic accuracy.

## 2 Methods

This study employed a retrospective design. The clinical data of 45 patients with DKA-AP who were admitted to the emergency department, gastroenterology department, endocrinology department, and critical care medicine department of Yangzhou University Affiliated Hospital between September 2013 and December 2024 were analyzed. To serve as a control group, DKA patients without AP (DKA-nonAP) with an equivalent number of cases during the same period were selected. The control group was matched to the DKA-AP group in terms of age, gender, and duration of diabetes, except for the absence of AP, in order to reduce the influence of confounding factors. The matching process was based on the propensity score matching method, whereby propensity scores were calculated based on demographics, medical history, symptoms, and laboratory parameters. DKA patients with similar scores were selected as the control group. The diagnosis of AP is based on the Guidelines for the Diagnosis and Treatment of Acute Pancreatitis in China (2021). AP diagnosis followed the 2021 Chinese guidelines for AP, which require at least two of the following: (1) characteristic abdominal pain; (2) serum amylase/lipase more than 3 times the upper limit of normal; (3) imaging findings consistent with AP (contrast-enhanced computed tomography or magnetic resonance imaging). The study was approved by the Institutional Ethics Committee of Yangzhou University Affiliated Hospital (2022-YKL3-06-004).

This study employed a mixed research methodology of retrospective cohort study and systematic review. This mixed research method was similar to that envisaged by Zhu et al. and Li et al. (3–7).

First retrospective cohort study included 90 patients (45 DKA-AP, 45 DKA-nonAP) admitted between 2013 and 2024. AP diagnosis followed the 2021 Chinese guidelines for acute pancreatitis. The idea of using the Chinese pancreatitis guidelines as a diagnostic criterion for AP is similar to the design of a Chinese DKA-AP study, which reduces study bias in China (8). Data were extracted from electronic health records using a standardized checklist, including demographics, medical history, symptoms, and laboratory parameters.

Yan Bo’s view was that every study should have a necessary literature review to complement the methodology or results (9). The literature related to the study of DKA-AP was then reviewed according to the PRISMA guidelines (10). As of February 2025, the terms “diabetic ketoacidosis” and “pancreatitis” were searched using the database PubMed. The search terms “diabetic ketoacidosis” and “pancreatitis” were selected as they pertain to the two diseases under investigation in this study. These terms were used to perform a search of the existing literature to identify studies that directly investigated the relationship between DKA and AP. Also, after the initial search, it was found that these two terms could cover most of the relevant studies. In addition, other synonyms or variants were not selected for the search because it was considered that too many search terms might introduce a large number of irrelevant literature and reduce the efficiency of the search.

Further additions can be made if subsequent studies reveal a need to expand the search scope. Only studies examining DKA-AP were included, including clinical randomized controlled studies, cohort studies and observational studies. This was done because we wanted to understand the progress of previous international research on identifying risk factors for DKA-AP. Articles may contain unifactorial analyses, multifactorial analyses, however case reports or case series alone are not sufficient to explore risk factors. Thus case reports and review articles that lacked exploration of risk factors were excluded. We ran backtracking methods applied to the included articles to capture potential literature. The method of performing systematic search and reverse tracking was informed by the method of Zhao et al., Ma et al., and Liang et al. (11–13), who argued that the heterogeneity of randomized controlled studies is the smallest of all clinical study types and should be considered first, and that the use of reverse tracking method may compensate for the shortcomings in the search formula. The search was limited to search terms appearing in the title, abstract and keywords to accurately screen the literature studying DKA-AP. Meanwhile, only English literature was included to ensure the quality of literature and language consistency. In addition, the retrieved literature was preliminarily screened to exclude literature that was not directly related to the study of DKA-AP, such as literature that only studied a single disease, DKA or AP, and did not address the association between the two.

Normality of continuous variables was assessed using Shapiro–Wilk tests. Non-normally distributed variables were analyzed with non-parametric tests (Mann–Whitney U test), while normally distributed variables were analyzed using t-tests. Categorical variables used Pearson’s χ^2^ test. For categorical variables with expected cell counts <5 in >25% of cells, Fisher’s exact test was applied. Multivariable logistic regression identified independent risk factors.

ROC assessed predictive performance (14). Variables included in the ROC analysis were selected based on three criteria: (1) significant association with DKA-AP in univariate analysis (*p* < 0.05) (2) clinical relevance supported by literature review; and (3) predefined cutoffs from prior studies (TG and TC). Optimal cutoffs were determined by maximizing Youden’s Index. To compare the discriminatory performance of individual variables and the combined model, DeLong’s test was employed, a non-parametric method for comparing the area under the curve (AUC) of two or more correlated ROC curves. This test accounts for the paired nature of the data and was robust to non-normal distributions. Statistical comparisons were performed using the pROC package in R (version 4.3.1). This idea was similar to the idea of Yang et al. (15).

Optimal cutoffs for ROC analysis were determined by maximizing Youden’s Index (16). Positive predictive value (PPV) and negative predictive value (NPV) (17) were calculated using the following formulas (Equations 1, 2).


(1) $$PPV=\frac{\mathrm{True positives}}{\left(\mathrm{True positives}+\mathrm{False positives}\right)}$$

(2) $$NPV=\frac{\mathrm{True negatives}}{\left(\mathrm{True negatives}+\mathrm{False negatives}\right)}$$

Analyses were performed using SPSS 25.0. Statistical significance was set at bilateral *p* < 0.05.

## 3 Results

### 3.1 Baseline characteristics

A total of 90 patients diagnosed with DKA were included in the study, comprising 45 cases with AP and 45 cases without AP. The DKA-AP group had higher BMI [26.30 (22.15–28.40) vs. 23.20 (20.70–25.35) kg/m^2^, *p* = 0.031], hyperlipidemia prevalence (26.7% vs. 4.4%, *p* = 0.004), and abdominal pain duration (3 vs. 0 days, *p* < 0.001). Significant differences were observed in hematocrit (HCT), red blood cell count (RBC), pH, bicarbonate (HCO₃^−^), glucose (Glu), total cholesterol (TC), triglycerides (TG), and amylase (AMY) (*p* < 0.05; Table 1). Some of laboratory examination data demonstrated no statistically significant differences in the analyzed parameters between the DKA-AP and DKA-nonAP groups. Specifically, there were no notable disparities observed in white blood cell count (WBC, 10^9^/L), neutrophil count (NEUT, 10^9^/L), creatinine (Cr, μmol/L), urea (mmol/L), lactate (Lac, mmol/L), glycated hemoglobin (HbA1c, %), fasting C-peptide (pmol/L), 2-h postprandial C-peptide (pmol/L), sodium (Na^+^, mmol/L), potassium (K^+^, mmol/L), calcium (Ca^2+^, mmol/L), high-density lipoprotein (HDL, mmol/L), low-density lipoprotein (LDL, mmol/L), alanine aminotransferase (ALT, U/L), aspartate aminotransferase (AST, U/L), or total bilirubin (TBIL, mmol/L) levels between the two groups. These findings suggested comparable laboratory profiles in both cohorts regarding the measured analytes.

**Table 1:** Comparative analysis of the basic conditions of the two groups.

Indicator	Subgroup	DKA-AP	DKA-nonAP	Statistical effect size	*p*-value
General demographic characteristics					
Sex^a^	Male	34 (75.60%)	27 (60.0%)	2.493	0.114
	Female	11 (24.40%)	18 (40.0%)		
Age (years)^b^		39.89 ± 10.74	39.11 ± 14.31	0.292	0.771
BMI (kg/m^2^)^c^		26.30 [22.15–28.40]	23.20 [20.70–25.35]	−2.155	0.031
Medical history					
Smoking history^a^	Yes	15 (33.33%)	11 (24.40%)	0.865	0.352
	No	30 (66.67%)	34 (75.60%)		
Drinking history^a^	Yes	12 (26.70%)	8 (17.80%)	1.029	0.31
	No	33 (73.30%)	37 (82.20%)		
Hypertension history^a^	Yes	8 (17.80%)	7 (15.60%)	0.08	0.777
	No	37 (82.20%)	38 (84.40%)		
Diabetes history^a^	Yes	30 (66.67%)	27 (60.00%)	0.431	0.662
	No	15 (33.33%)	18 (40.00%)		
Diabetes type^a^	Type 1	10 (22.2%)	17 (37.8%)	2.593	0.107
	Type 2	35 (77.8%)	28 (62.2%)		
Hyperlipidemia history^a,d^	Yes	12 (26.70%)	2 (4.40%)	8.459	0.004
	No	33 (73.3%)	43 (95.60%)		
Gallstone disease history^a,d^	Yes	3 (6.70%)	1 (2.20%)	1.047	0.616
	No	42 (93.30%)	44 (97.80%)		
Symptoms					
Nausea and vomiting^a^	Yes	21 (46.70%)	30 (66.67%)	3.665	0.088
	No	24 (53.30%)	15 (33.33%)		
Abdominal pain^a^	Yes	35 (77.80%)	6 (13.30%)	37.675	<0.001
	No	10 (22.20%)	39 (86.70%)		
Polydipsia, polyuria, polyphagia, and weight loss^a^	Yes	10 (22.20%)	15 (33.33%)	1.385	0.239
	No	35 (77.80%)	30 (66.67%)		
Fever^a,d^	Yes	4 (8.90%)	1 (2.20%)	0.847	0.375
	No	41 (91.10%)	44 (97.80%)		
Consciousness disorder^a,d^	Yes	6 (13.33%)	4 (8.89%)	0.45	0.502
	No	39 (86.67%)	41 (91.11%)		
Abdominal pain duration (days)^c^		3 [1–5]	0	−6.636	<0.001
Laboratory examination data					
HCT (%)^b^		46.76 ± 5.24	44.46 ± 4.36	2.256	0.027
RBC (10^9^/L)^b^		5.29 ± 0.67	4.91 ± 0.51	3.013	0.003
HB (g/L)^c^		164 [148–186]	154.0 [141.0–165.5]	−2.39	0.017
pH^c^		7.11 [7.015–7.20]	7.20 [7.14–7.23]	2.884	0.004
CO_2_CP (mmol/L)^c^		16.00 [11.00–25.50]	24 [15–27]	2.215	0.027
HCO_3_^−^ (mmol/L)^c^		5.7 [3.4–9.7]	9.7 [4.3–11.7]	2.017	0.044
Glu (mmol/L)^c^		29.73 [23.30–36.58]	25.20 [20.23–30.51]	−2.324	0.02
TC (mmol/L)^c^		7.71 [5.60–11.66]	4.86 [4.22–6.52]	−4.018	<0.001
TG (mmol/L)^c^		11.90 [4.71–15.84]	1.60 [0.89–3.64]	−6.282	<0.001
AMY (U/L)^c^		215.0 [80.5–540.0]	92.0 [68.0–182.5]	−2.986	0.003

^aPearson’s χ2 test; bt-test; cMann–Whitney U test; dFisher’s exact test. p-value is the result of two-sided test, and p < 0.05 indicates that the difference is statistically significant. DKA-AP, Diabetic ketoacidosis with acute pancreatitis; DKA-nonAP, Diabetic ketoacidosis without acute pancreatitis; p-value, Probability value; BMI, Body mass index; HCT, Hematocrit; RBC, Red blood cell; HB, Hemoglobin; pH, Potential of hydrogen; CO2CP, Carbon dioxide combining power; HCO3−, Bicarbonate; Glu, Glucose; TC, Total cholesterol; TG, Triglyceride; AMY, Amylase.^

A total of 90 patients diagnosed with DKA were included in the study, comprising 45 cases with AP and 45 cases without AP. The DKA-AP group had higher BMI (26.30 [22.15–28.40] vs. 23.20 [20.70–25.35] kg/m^2^, *p* = 0.031), hyperlipidemia prevalence (26.7% vs. 4.4%, *p* = 0.004), and abdominal pain duration (3 vs. 0 days, *p* < 0.001). Significant differences were observed in hematocrit (HCT), red blood cell count (RBC), pH value (pH), carbon dioxide combining power (CO₂CP), bicarbonate (HCO_₃_^−^), glucose (Glu), total cholesterol (TC), triglyceride (TG), and amylase (AMY) (*p* < 0.05; Table 1).

### 3.2 Multivariable logistic regression analysis

Multivariable logistic regression analysis was conducted on the indicators with significant differences in the univariate analysis, as illustrated in Table 2. The findings revealed that cholesterol, triglyceride levels, and the abdominal pain duration were independent predictors of DKA-AP. Nagelkerke R^2^ = 0.621 indicating that the model explained 62.1% of the variance.

**Table 2:** Logistic regression analysis of risk factors associated with DKA-AP.

Factor	B	SE	Wald	OR	*p*	95% CI
Abdominal pain duration	1.359	0.297	20.869	3.892	<0.001	2.173–6.972
TC (mmol/L)	0.375	0.100	14.116	1.455	<0.001	1.196–1.769
TG (mmol/L)	0.716	0.271	6.956	2.046	0.008	1.202–3.484

B, Regression coefficient; SE, Standard error; Wald, Wald statistic; OR, Odds ratio; *p*, *p*-value; 95% CI, 95% confidence interval; TC, Total cholesterol; TG, Triglyceride.

### 3.3 Comparative analysis of ROC curve

**Figure 1:**
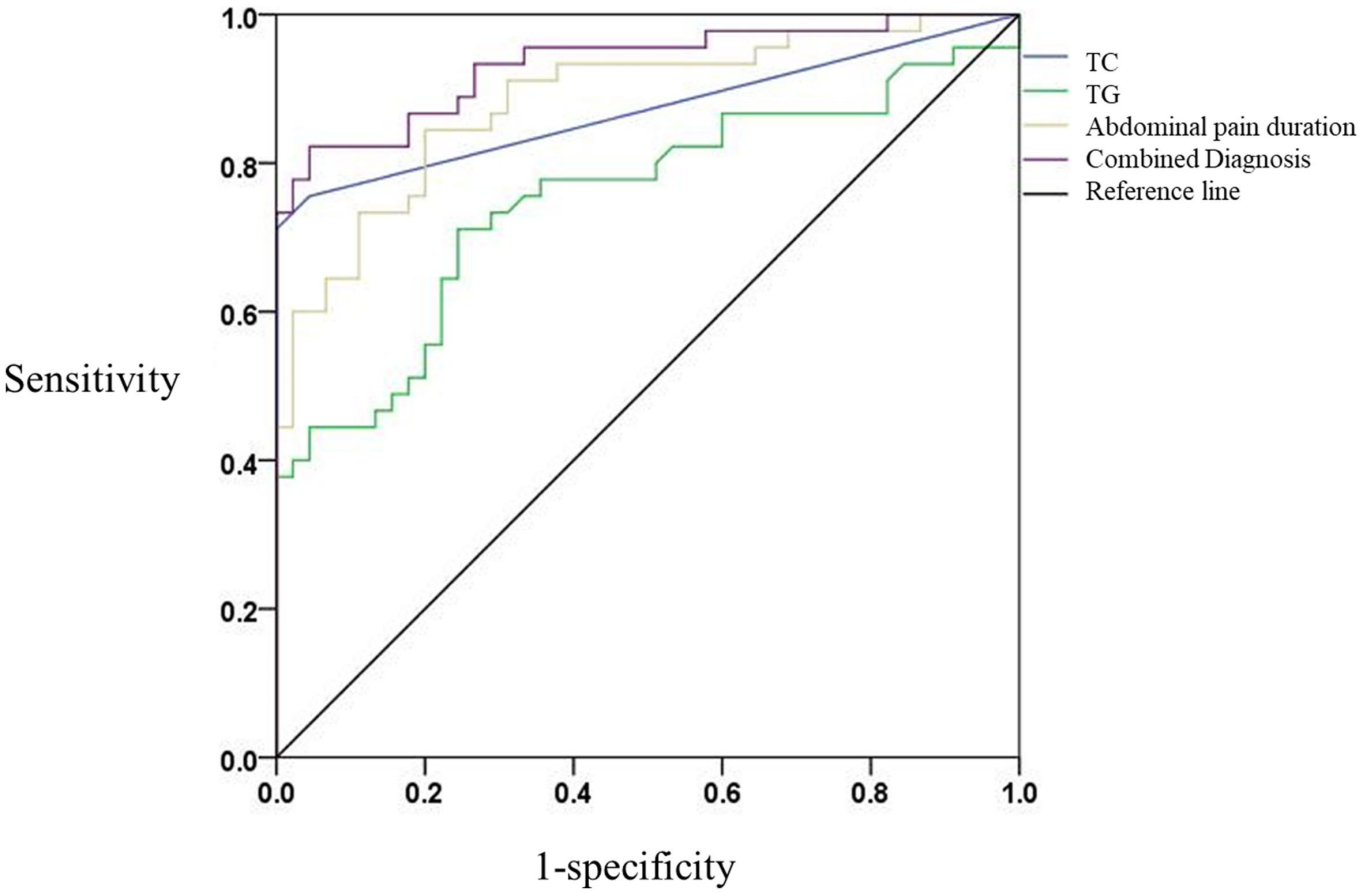
Comparison of ROC curves in subjects with different independent risk factors. Blue line: TC (AUC = 0.751, 95% CI: 0.648–0.855). Green line: TG (AUC = 0.884, 95% CI: 0.815–0.953). Yellow line: abdominal pain duration (AUC = 0.871, 95% CI: 0.791–0.951). Purple line: Combined model (AUC = 0.933, 95% CI: 0.881–0.986). The horizontal coordinate is the false positive rate (1-specificity) and the vertical coordinate is the true positive rate (sensitivity). The different curves have different meanings. The blue line is the TC predictive ROC curve for DKA-AP; the green line is the TG predictive ROC curve for DKA-AP; and the yellow line is the abdominal pain duration predictive ROC curve for DKA-AP; the purple line represents the ROC curve predicted by the combined diagnosis; the black line represents the reference line.

ROC analysis demonstrated strong predictive performance for individual variables and their combination. Using a TG cut-off of ≥11.90 mmol/L, the sensitivity and specificity for DKA-AP diagnosis were 84.4% and 80.0%, respectively, with a PPV of 81.0% and NPV of 83.7%. Similarly, a TC cut-off of ≥7.71 mmol/L achieved 71.1% sensitivity and 75.6% specificity (PPV 74.3%, NPV 72.5%). Abdominal pain duration ≥3 days showed 77.8% sensitivity and 86.7% specificity (PPV 85.2%, NPV 80.0%). The combined model exhibited the highest discriminative power (AUC = 0.933, 95% CI: 0.881–0.986), with 93.3% sensitivity and 73.3% specificity (PPV 80.6%, NPV 90.0%). ROC analysis showed high discriminative power (*p* < 0.05; Table 3; Figure 1).

**Table 3:** Area under the ROC curve with optimal cutoffs and predictive performance.

Variable	AUC (95% CI)	Cut-off	Sensitivity (%)	Specificity (%)	PPV (%)	NPV (%)	Youden’s Index
TC (mmol/L)	0.751 (0.648–0.855)	≥7.71	71.1	75.6	74.3	72.5	0.467
TG (mmol/L)	0.884 (0.815–0.953)	≥11.90	84.4	80.0	81.0	83.7	0.644
Abdominal pain duration (days)	0.871 (0.791–0.951)	≥3	77.8	86.7	85.2	80.0	0.644
Combined diagnosis	0.933 (0.881–0.986)	-	93.3	73.3	80.6	90.0	0.667

95% CI, 95% confidence interval; TC, total cholesterol; TG, triglyceride; PPV, positive predictive value; NPV, negative predictive value. Optimal cutoffs were determined by maximizing Youden’s Index. Combined diagnosis model includes TC, TG, and abdominal pain duration.

DeLong’s test showed that the combined model (TC + TG + abdominal pain duration) had a significantly higher AUC (0.933) than TC (0.751, *p* < 0.001), TG (0.884, *p* = 0.023) and abdominal pain duration (0.871, *p* = 0.015). Although the AUC of TG (0.884) was significantly higher than that of TC (0.751, *p* < 0.001), its specificity (80.0%) was lower than that of abdominal pain duration (86.7%, *p* = 0.035). The combined model, by integrating three significant variables, performed optimally in terms of sensitivity (93.3%) and negative predictive value (90.0%), suggesting its clinical predictive value in assessing the diagnosis of DKA-AP (Table 3; Figure 1).

### 3.4 Literature review

**Figure 2:**
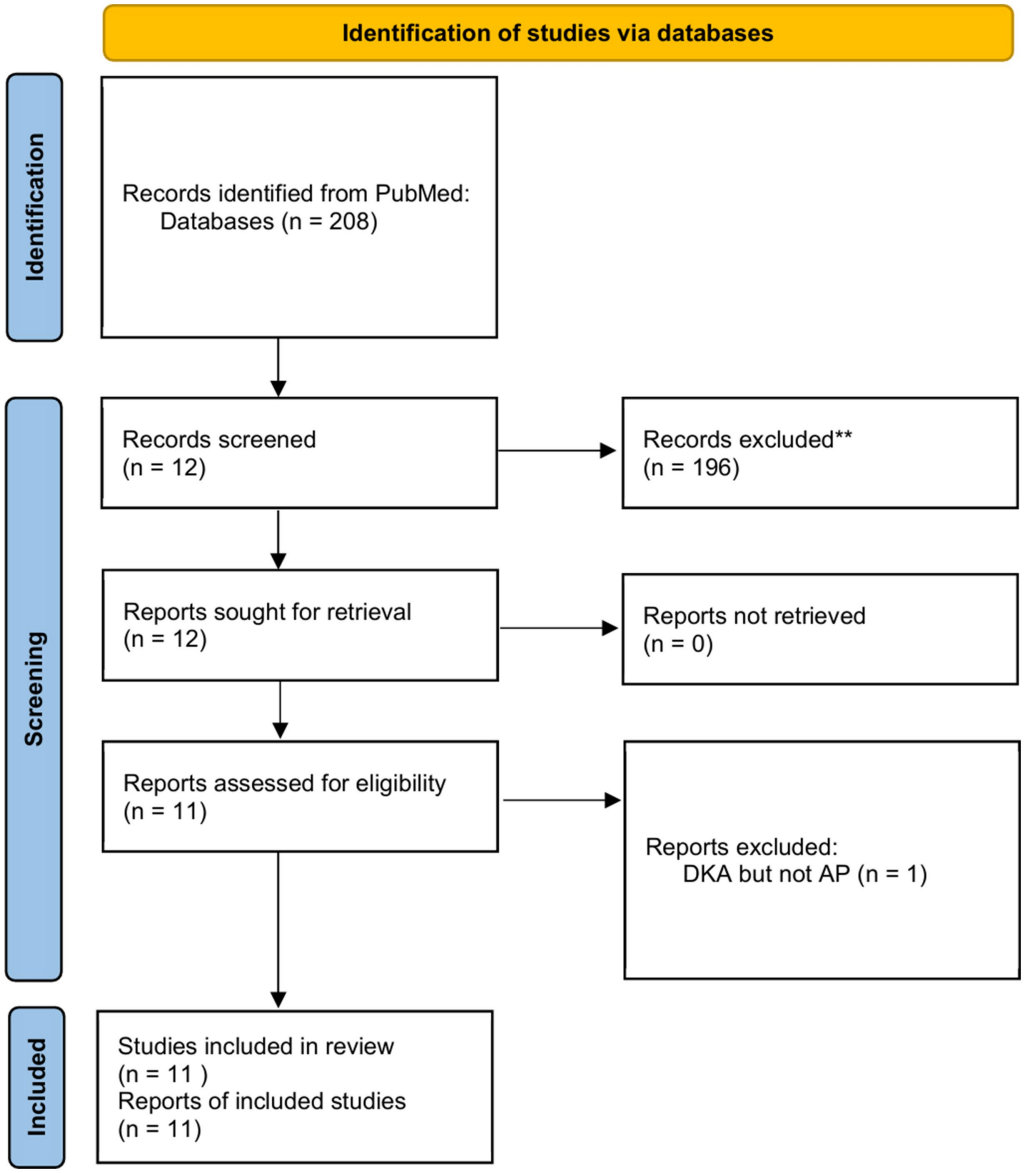
Flowchart of literature selection using the PRISMA guidelines.

After a systematic search in the PubMed database (Figure 2), we found 11 clinical studies investigating DKA-AP (1, 8, 18–26). Cumulatively, 1,851 patients were studied, including three interventional studies, 1 genetic observational study, and 7 cohort studies exploring risk factors (Table 4). Of the 7 cohort studies exploring risk factors, 2 studies analyzed risk factors using area under the ROC curve, 2 studies analyzed risk factors using a multifactorial approach, and the remaining 3 used a univariate or descriptive method of Analysis.

**Table 4:** Review of 1,851 cases of clinical study of DKA-AP.

No.	References	Design	Population	Sex (M/F)	Age (years)	Risk factors	Treatment
1	Nair et al. (1)	Prospective observational study	DKA-AP vs. DKA-nonAP	8/3 vs. 42/44	53.0 ± 16.0 vs. 50.48 ± 22.93	Transient and profound hyperlipidemia, acidosis and hyperglycemia. Elevation of serum lipase and amylas.	NA
2	Wang et al. (18)	Retrospective cohort study	DKA-AP vs. nonDKA-AP	33/4 vs. 74/29	37.7 (10.5) vs. 40.3 (9.5)	DKA-Ap has a higher Ranson standard score[(3.97 vs. 2.88; *p* = 0.001)] and Acute Physiology and Chronic Health Evaluation II (APACHE II) score[(7.70 vs. 5.39; *p* = 0.007)] than nonDKA-AP.	NA
3	Yuan et al. (19)	Retrospective cohort study	DKA-AP vs. nonDKA-AP	29/19 vs. 37/23	42.52 ± 15.29 vs. 47.81 ± 15.57	Comorbidity histories of diabetes mellitus [adjusted OR = 55.128, 95% CI(10.469–290.306), *p* < 0.01)] And not alert [(adjusted OR = 32.547, 95% CI(4.701–225.322), *p* < 0.01]	NA
4	Huang et al. (20)	Retrospective observational study	DKA-AP-HTG	5/1	24–36	c.12614C>T(p.Pro4205Leu) in *APOB*, c.160G>C(p.Glu54Gln) in *CILP2*, and c.1199C>A(p. Ala400Glu) in *PEPD*.	NA
5	Wei et al. (21)	Prospective cohort study	DKA-AP	NA	NA	Abdominal distension, abdominal pain, body temperature, blood sugar, HbA1c and blood amylase, AMS, blood ketones, PCT, TG, PTX–3, CX3CL1	Insulin pump combined with ustekin vs. insulin pump
6	Yin et al. (22)	Prospective cohort study	DKA-AP	18/14 vs. 17/15	51.03 ± 5.21 vs. 50.98 ± 5.19	Albumin (mmol/L), PCT (U/L), CRP (mmol/L), TNF-α (ng/mL), PTX-3 (ng/mL), AMS (U/L),IgG (g/L), IgM (g/L) and IgA (g/L)	Nutritional support combined with insulin vs. insulin
7	Fu et al. (23)	Retrospective cohort study	DKA-AP vs. nonDKA-AP	22/5 vs. 78/31	31 (25–35) vs. 50 (35–70)	Blood glucose [26.1 (21.8–35.6) vs. 12.6 (9.6–17.6) mmol/L, z = −7.204, *p* < 0.001; AUC 0.949, 95% CI(0.909–0.988)]. When the blood glucose concentration cut-off value > 21.75 mmol/L was used to diagnose DKA-AP, the sensitivity was 81.5%, the specificity was 94.5%, the positive predictive value was 78.6%, the negative predictive value was 95.4%, and the accuracy was 91.9%.	NA
8	Ma et al. (8)	Prospective cohort study	DKA-AP vs. DKA-nonAP	19/6 vs. 77/59	30 (26.50,14.25) vs. 37 (29.00,53.00)	Serum triglyceride (mmol/L) [AUC 0.93, 95% CI(0.875–0.985)], Serum cholesterol (mmol/L) [AUC 0.86, 95% CI (0.768–0.951)] and pH [AUC 0.821, 95% CI(0.737–0.905)]. When Serum triglyceride cut-off > 10.52 mmol/L was used to diagnose DKA-AP, the sensitivity was 80%, the specificity was 93.2%, the positive predictive value was 60.7%, and the negative predictive value was 94%. When Serum cholesterol cut-off > 9.03 mmol/L was used to diagnose DKA-AP, the sensitivity was 72%, the specificity was 91.7%, the positive predictive value was 62%, and the negative predictive value was 94.7%. When pH cut-off > 7.214 was used to diagnose DKA-AP, the sensitivity was 75.7%, the specificity was 72%, the positive predictive value was 42.7%, and the negative predictive value was 93.5%.	NA
9	Khan et al. (24)	Retrospective cohort study	DKA-AP vs. DKA-nonAP	67/17 vs. 521/331	42.7 ± 12.8 vs. 35.3 ± 14.5	Age [Odds ratio(OR) = 1.04, 95% CI(1.003–1.070), *p* = 0.02] and total cholesterol levels [OR = 1.20, 95%CI (1.050–1.400), *p* < 0.01]	
10	Li and Li (25)	Prospective cohort study	DKA-AP vs. nonDKA-AP	5/2 vs. 15/3	26.0 vs. 47.5	Uric acid concentration[442 (352.00–698.00) vs. 297.5 (193.75–459.00) umol/L, *p* = 0.041] and poor glycemic control[7 (100%) vs. 5 (27.78%), *p* = 0.02]	
11	Zhang and He (26)	RCT	DKA-AP	32/21 vs. 28/25	49.55 ± 6.83 vs. 48.75 ± 7.14	Abdominal pain (30.2% vs. 13.2%), nausea and vomiting (18.9% vs. 5.7%), excessive drinking and urination (26.4% vs. 11.3%), diarrhea (17.0% vs. 3.8%), impaired consciousness (15.1% vs. 1.9%) and hypotension or shock (11.3% vs. 0%).	Dexamethasone vs. placebo

The data in the table are expressed as mean ± standard deviation if normally distributed or median (interquartile spacing) if skewed. Values are non-parametric test statistics, values are parametric test statistics, and X^2^ is the chi-square test statistic. *p*-value is the result of two-sided test, and *p* < 0.05 indicates that the difference is statistically significant. Age is presented as mean, median or maximum. The order of priority for risk factor presentation was: predictors after ROC curve analysis > multifactorial analysis > unifactorial analysis > clinical symptoms.

The results indicated the presence of numerous significant unifactorial differences in clinical symptoms, clinical assessment scores, laboratory tests and imaging in cases where DKA and AP developed concurrently. These differences are not universal and the main reason may be that the population investigated is so small that there is a large single-center bias. However, blood glucose values, total cholesterol levels, age, and pH derived from ROC curve analysis can alert the first clinician in the clinic. Khan et al. (24) showed that the younger the age the more likely to be comorbid with AP in patients with DKA.

## 4 Discussion

### 4.1 Key results

The aim of this study was to investigate the risk factors for DKA-AP and to assess the predictive efficacy of the identified risk factors. The results of the study showed significant differences between the DKA-AP group and the DKA-nonAP group in a number of indices, with the differences in BMI, history of hyperlipidemia, duration of abdominal pain, and lipid profiles (TC, TG) being particularly prominent. TC, TG levels and duration of abdominal pain were identified as independent predictors of DKA-AP by multifactorial analysis, and ROC curve analysis showed that the combination of these factors had good predictive performance (AUC = 0.933, sensitivity = 93.3%, specificity = 73.3%). In addition, patients with DKA had elevated levels of TG (>11.97 mmol/L), reflecting disturbed lipid metabolism, which may trigger AP through free fatty acid-mediated pancreatic injury. Notably, abdominal pain duration over 3 days became a new predictor. This is in line with the aim of the study and provides an important basis for early clinical recognition of DKA-AP.

The identification of TC ≥ 7.71 mmol/L, TG ≥ 11.90 mmol/L, and abdominal pain duration ≥ 3 days as predictors enables clinicians to prioritize lipid-lowering therapies and expedite pancreatic imaging in high-risk DKA patients. This approach may reduce complications such as pancreatic necrosis or organ failure, aligning with guidelines for early intervention in hypertriglyceridemia-associated pancreatitis.

The high PPV and NPV of TG (81.0% and 83.7%) and abdominal pain duration (85.2% and 80.0%) suggest their utility as reliable screening tools in emergency settings. Clinicians should prioritize TG testing and abdominal pain assessment in DKA patients to expedite AP diagnosis.

### 4.2 Limitations

There are some limitations to this study. Regarding the sample size, only 90 patients were included during the 11-year period, a small sample size, which limits the feasibility of subgroup analyses and may not accurately reflect the situation of patients in different subgroups. For example, it is difficult to carry out in-depth analyses of the differences in risk factors in different gender and age subgroups, which limits the precision of the study results.

On potential confounders, there were unmeasured confounders that could have affected the results, although some were considered in the study design. Patient dietary composition and physical activity were important potential confounders. Chronic high-triglyceride diets may increase the risk of AP development and are also associated with the development and poor control of DKA. Inadequate exercise may lead to metabolic disturbances that promote the development of DKA and AP. As this information was not collected in this study, it could not be adjusted in the analyses, which may have led to biased results. This bias may make the association between risk factors and DKA-AP overestimated or underestimated, interfering with the judgment of the true relationship.

In addition, this study was a single-center retrospective study with single-center bias. The single-center patient population has limitations in terms of geography, ethnicity and medical level, which may not be representative of the wider patient population and affect the generalizability of the study results.

### 4.3 Interpretation

In terms of the achievement of the study objectives, this study successfully targeted TC, TG and abdominal pain duration as the key predictors of DKA-AP. This result is important for achieving the goal of exploring risk factors and provides a key reference for early clinical identification of DKA-AP, which will help clinicians to take timely interventions to improve patients’ prognosis.

When compared with similar studies, the results of the present study present similarities and differences. In terms of hypertriglyceridemia as a risk factor for DKA-AP, the findings of several studies are consistent. Nair et al. conducted a prospective study on 100 patients with DKA and found that 11% of them had concomitant AP, and among them, 4 patients with AP had severe hypertriglyceridemia, and their triglyceride levels returned to near normal after remission of the DKA. Nair’s results strongly suggest a strong association between hypertriglyceridemia and DKA-AP (1). Nair’s results strongly demonstrated the strong association between hypertriglyceridemia and DKA-AP (1). Huang et al. (20) conducted a study on 6 patients with DKA and hypertriglyceridemia, and the findings of the present study showed similarities. Huang et al. (20) studied six patients with DKA, hypertriglyceridemia, and the AP triad and found that the patients’ triglyceride levels were extremely high, with a mean of 3282.17 ± 2975.43 mg/dL, further supporting this view. In addition, Yuan et al. showed a significantly higher incidence of hypertriglyceridemia in AP patients with combined DKA compared to AP patients without DKA. Fu et al. (23) also demonstrated that patients in the DKA group had significantly higher triglyceride levels than those in the non-DKA group. Together, these studies confirm the important role of hypertriglyceridemia in the pathogenesis of DKA-AP.

However, there were differences between the present study and other studies regarding the role of factors such as BMI and age. BMI was not identified as an independent risk factor in the present study, whereas Khan et al. (24) showed that BMI was significantly higher in patients with DKA-AP than in patients with DKA-nonAP. This may be due to the geographic and ethnic differences in the sample of this study, which was relatively small, resulting in an inadequate ability to detect the effect of BMI. Also, the different ways of measuring and categorizing BMI in the study methodology may have contributed to the difference in results.

Regarding age, the present study did not find any significant association between age and DKA-AP, whereas Khan et al. showed that age was a significant risk factor for DKA-AP and that patients with DKA-AP were older (24). However, a study by Fu et al. (23) found that among patients with type 2 diabetes combined with AP, those in the DKA group were younger than those in the non-DKA group. This difference may be related to the age range of patients included in each study, type of disease and study design. The age range of patients included in this study may have been narrower, making the effect of age difference on the results not fully apparent; and the different selection criteria for DKA and AP patients in different studies may have led to inconsistencies in the age-DKA-AP relationship. These could interfere with the results of studies of the association of the age factor with DKA-AP.

In addition, other studies have addressed some aspects that were not explored in depth in this study. For example, Wang et al. (18) investigated the effect of DKA on the clinical course and severity scores of hypertriglyceridemia-induced pancreatitis (HP), and found that DKA led to increased acute kidney injury and higher chance of ICU admission in patients with HP. Wang’s results are different from the risk factors that were focused on in the present study, but they also suggest that the different aspects of the disease need to be considered comprehensively in the assessment of DKA-AP. The results of Wang’s study are different from the present study but suggest that different aspects of the disease need to be considered together when assessing DKA-AP. Other studies have focused on the effect of treatment modalities on patients with DKA-AP. Wei et al. (21) found that insulin pumps combined with ustekin therapy reduced PCT, TG, and other markers in patients. Yin et al. (22) demonstrated that nutritional support combined with insulin therapy improved serum protein levels and reduced inflammatory response in patients. These studies provide a reference for the clinical management of DKA-AP from a therapeutic point of view, and also show that when interpreting the results of the risk factors in this study, it is necessary to make a comprehensive judgment in combination with the impact of different treatment modalities on the disease.

Multiple analyses were conducted in the study, and although methods such as multivariable logistic regression analyses were used to identify independent risk factors and ROC curves were used to assess the predictive performance, there may be some limitations and uncertainties in the different analytical methods. When combining the results of multiple analyses, there may also be contradictory or difficult-to-interpret situations.

Although this study has achieved some results in exploring the risk factors of DKA-AP, clinicians should be cautious in applying the results of this study due to the limitations of the study and the differences with similar studies. The results cannot be directly generalized to all DKA patients, but should be used in conjunction with the individual patient’s condition, the characteristics of the region, and other clinical evidence to formulate a more accurate and effective diagnosis and treatment plan. Future studies need to expand the sample size, conduct multicenter studies, and include more potential confounding factors to further validate and improve the findings of this study.

## 5 Conclusion

Elevated TC, TG, and prolonged abdominal pain duration are critical predictors of DKA-AP. These factors enhance diagnostic accuracy and guide targeted management, reducing morbidity in high-risk populations. Future multi-center studies with larger cohorts are warranted to validate these predictors and explore additional risk factors, such as genetic or lifestyle-related variables. At the same time, a prospective study design can be adopted to track the changes of patients’ conditions in depth and establish a better risk prediction model to provide a stronger basis for early clinical diagnosis and intervention.

## Data Availability

The original contributions presented in the study are included in the article/supplementary material, further inquiries can be directed to the corresponding authors.
